# Maximizing the Impact of Voluntary Medical Male Circumcision for HIV Prevention in Zambia by Targeting High-Risk Men: A Pre/Post Program Evaluation

**DOI:** 10.1007/s10461-022-03767-6

**Published:** 2022-07-28

**Authors:** M. Lukobo-Durrell, L. Aladesanmi, C. Suraratdecha, C. Laube, J. Grund, D. Mohan, M. Kabila, F. Kaira, M. Habel, J. Z. Hines, H. Mtonga, O. Chituwo, M. Conkling, P. J. Chipimo, J. Kachimba, C. Toledo

**Affiliations:** 1 Jhpiego, Baltimore, MD USA; 2 Jhpiego, Lusaka, Zambia; 3 Division of Global HIV and TB, Center for Global Health, Centers for Disease Control and Prevention, Atlanta, GA USA; 4 Johns Hopkins Bloomberg School of Public Health, Baltimore, MD USA; 5 Division of Global HIV and TB, Center for Global Health, Centers for Disease Control and Prevention, Lusaka, Zambia; 6 Ministry of Health, Lusaka, Zambia; 7 Jhpiego, 1615 Thames Street, MD 21231 Baltimore, USA

**Keywords:** Male circumcision, Demand creation, Economic compensation, Zambia, Human-centered design

## Abstract

A well-documented barrier to voluntary medical male circumcision (VMMC) is financial loss due to the missed opportunity to work while undergoing and recovering from VMMC. We implemented a 2-phased outcome evaluation to explore how enhanced demand creation and financial compensation equivalent to 3 days of missed work influence uptake of VMMC among men at high risk of HIV exposure in Zambia. In Phase 1, we implemented human-centered design-informed interpersonal communication. In Phase 2, financial compensation of ZMW 200 (~ US$17) was added. The proportion of men undergoing circumcision was significantly higher in Phase 2 compared to Phase 1 (38% vs 3%). The cost of demand creation and compensation per client circumcised was $151.54 in Phase 1 and $34.93 in Phase 2. Financial compensation is a cost-effective strategy for increasing VMMC uptake among high-risk men in Zambia, and VMMC programs may consider similar interventions suited to their context.

## Introduction

Male circumcision is a cost-effective HIV prevention intervention that has been shown to reduce the risk of HIV transmission to men through heterosexual sex by approximately 60% [1–4]. Adult male circumcision also provides an opportunity to increase the number of men getting tested for HIV, as well as linking HIV positive men to treatment, contributing to the UNAIDS’ targets of 90-90-90 for HIV prevention and treatment [5]. As of December 2019, over 22 million voluntary medical male circumcisions (VMMCs) had been conducted in 15 priority sub-Saharan African countries [6]. Although this represents a significant achievement and progress towards the 2020 goal of an additional 25 million circumcisions for HIV prevention, male circumcision prevalence in the highest risk age groups (15–29 years) has lagged and most of the 15 priority countries have struggled to reach 80% male circumcision coverage targets. Interventions and efforts to reach men who have been hesitant to adopt VMMC, such as innovative demand creation strategies, are urgently needed. Zambia has a generalized HIV epidemic with heterosexual sex as the primary mode of transmission. According to the 2016 Zambia Population-based HIV Impact Assessment [7], 11.4% of adults aged 15–49 years are infected with HIV (14.3% of women and 8.3% of men) and annual incidence of HIV among men 15–59 years is 0.29% [7]. With a severe generalized HIV epidemic and a low male circumcision prevalence, Zambia adopted VMMC as part of a combination HIV prevention strategy and launched the VMMC program in 2007. Between 2008 and 2018, more than 2.4 million circumcisions were conducted [6]. This achievement is attributed to the rapid expansion and endorsement of the VMMC program by the Ministry of Health (MoH) and other partners. In 2018, 65% of circumcisions performed in Zambia were in men aged 19 years or younger and 30% were in men between 20 and 49 years old [8]. Studies show that circumcising HIV-negative men, 20–34 years old, at the highest risk of HIV acquisition through heterosexual intercourse (e.g., men in serodiscordant relationships, men with multiple sexual partners, men who engage in transactional sex, or have a history of STI diagnosis and/or symptoms) achieves the greatest effectiveness for VMMC programs [9, 10].

Although circumcising men at highest risk of HIV acquisition (referred to from here on as high-risk men) achieves the most immediate impact on the epidemic, finding and engaging these men in prevention services like VMMC remains challenging [11, 12]. Strategies to increase demand through identifying and engaging high-risk men who have not taken up VMMC are needed. Since the roll-out of the VMMC program began, a wide range of demand creation strategies have been implemented with varying effectiveness aiming at reducing barriers to VMMC and increasing uptake [12].

A well-documented barrier to VMMC is the cost of transport to travel to the clinic for surgery, as well as for at least one in-person, follow-up visit 48 h after surgery [13]. In addition to these costs, there may be financial loss due to time taken from work to undergo and recover from circumcision, which requires a minimum of 2 days of rest immediately after surgery and light physical exertion for at least 7 days in total [14]. Given these costs, the long-term benefits of VMMC may not outweigh the immediate opportunity cost particularly in households where men are the primary income generators. Offering financial incentives may provide a solution to address these barriers. VMMC is a one-time surgical intervention offering partial lifelong HIV prevention benefits making it a good candidate for economic compensation—a one-time financial incentive provided to VMMC clients to cushion the financial loss experienced when one undergoes VMMC would likely motivate men who are hesitant to get circumcised due to financial constraints.

Researchers have been investigating the use of incentives and economic compensation in HIV research in developing countries for several years. Results from systematic reviews and meta-analyses of studies on the effectiveness of economic compensation and incentives to increase VMMC uptake among men in sub-Saharan Africa found that economic compensation interventions had a positive impact on VMMC uptake and indicated that cash reimbursements for transportation and food vouchers to partially compensate for wage loss were effective at motivating older men who had already been interested in circumcision to get circumcised [15–17]. In the Nyanza region of Kenya, economic compensation offered in the form of food vouchers valued at ~ US $2.50, $8.75, or $15 increased VMMC uptake among men receiving ~ $8.75 and $15 vouchers. These amounts corresponded to transportation costs to the clinic and a portion of lost wages [18]. Food vouchers of similar value increased VMMC in another study in Kenya [19]. In Orange Farm, South Africa, where VMMC coverage stagnated around 55% coverage during 2010–2015, a demand creation strategy combining motivational interviewing with a cash incentive (~ $17.00) was able to achieve VMMC saturation (≥ 80% coverage) over 3 months among a random subset of men aged ≥ 18 years [20]. Another study in South Africa examined the effect of sending postcards to men offering a conditional cash transfer of ~ US $10 for attending a VMMC counseling session. This study found that the odds of getting circumcised among men receiving postcards were approximately four times higher (OR 3.8) than men not receiving postcards. In addition, they found that sending a postcard that included a message communicating a “challenge”, in addition to the cash transfer, had the largest effect (OR 5.3) [21]. However, other studies in Tanzania and Kenya involving raffles or lotteries of food vouchers, money, or smartphones did not lead to increases in VMMC uptake [19, 22]. Results revealed that clients found raffles and lotteries undesirable and even suspicious [22]. A mixed methods systematic review of the effectiveness of demand creation interventions for VMMC in sub-Saharan Africa found that demand creation strategies offering financial incentives produced the greatest impact on VMMC uptake [11].

We evaluated the phased implementation of an enhanced demand creation strategy for high-risk men developed using the behavioral-psychographic approach [23] with the addition of financial compensation. The main aims of this evaluation were to (1) evaluate the effectiveness of an enhanced demand creation strategy of targeted messaging among high-risk men aged 18 years and older at non-traditional VMMC recruitment sites with and without financial compensation on VMMC uptake (circumcision within 3 months of recruitment) and to (2) assess the cost of the enhanced demand creation strategy and financial compensation.

## Methods

### Study Design

**Fig. 1:**
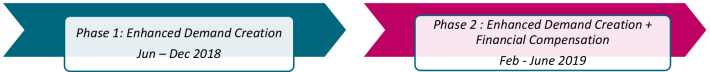
Evaluation phases

The evaluation was designed as a pre-post quantitative adequacy assessment of a phased implementation of an enhanced demand creation activity alone (Phase 1, June–December 2018) and accompanied by financial compensation of ZMW 200 (~ $17) cash, equivalent to 3 days of missed work and transport costs (Phase 2, February–June 2019) targeted at high-risk men 18 years and older. The primary outcome was the proportion of enrolled high-risk men undergoing VMMC at the participating facilities during each phase of the program (Fig. 1).

### Study Setting and Sites

Seven VMMC sites located in Lusaka and Mazabuka districts were selected to take part in the evaluation. These two districts were selected for this evaluation so that men from urban and peri-urban settings, daily-wage workers, and seasonal, plantation-based workers could be included, as these are all groups that may be at increased risk of HIV infection.

### Participants

Uncircumcised adult men aged 18 years and older, living within the catchment areas of selected VMMC sites and self-reporting at least one HIV risk factor (RF) were eligible to participate in the evaluation. High-risk was defined as a self-report of at least one of six pre-defined HIV RFs *in the past 6 months*: (i) Treatment for a sexually transmitted infection (STI) or symptoms of an STI, including current STI) (ii) participation in transactional sex (e.g., buying or selling sex for money, food, or favors); (iii) had sex with HIV-positive primary sexual partner (as defined by the participant); (iv) had more than 2 concurrent sexual partners; (v) sexual intercourse when the participant or his partner were intoxicated; (vi) used illegal drugs (e.g., marijuana, dagga, heroin, ecstasy). Men who did not meet the above criteria or refused to participate were excluded from the evaluation.

### Sample Size Considerations

The primary driver of the power calculations was the minimum detectable difference in the proportion of high-risk men enrolled who undergo VMMC at the catchment facilities between Phase 1 and Phase 2 of the program. Due to lack of previous examples, varying power calculations for a sample size of 8,000 in each phase were calculated using two values of intraclass cluster co-efficient (ICC) − 0.05 and 0.1. The power calculations were run using the clusterPower package in R. The type I error rate was assumed to be 0.05 with 20 mobilizers (number of clusters) and 400 enrollees per mobilizer.

### Intervention

An enhanced demand creation strategy with financial compensation targeted at high-risk men 18 years and older (Table 1) was designed, implemented, and evaluated. The enhanced demand creation strategy used a human centered design (HCD) approach and was implemented by community health promotors at targeted venues in the community. Targeted venues included bars, brothels, workplaces (e.g., sugar plantations), higher institutions/university and STI clinics. The HCD approach for demand creation uses a segmentation tool to categorize men by their attitude about and motivation for getting circumcised, and then provide client-centered information to address known barriers to seeking the procedure in that behavioral segment [23].

**Table 1:** Components of the enhanced demand creation strategy

Components	Enhanced demand creation
Demand creation strategy	One-on-one Human Centered Design (HCD) approach conducted by community health promotors at non-traditional targeted venues in the community
	Mobilization activities occurred selectively during the day and night in order to reach males who are difficult to access during regular daytime mobilization hours
Target population	Men ≥ 18 years, uncircumcised, and self-report one of the following risk behaviors in ≤ 6 months: (i) Treatment for a sexually transmitted infection (STI) or symptoms of an STI, including current STI; (ii) participation in transactional sex (e.g., buying or selling sex for money, food, or favors); (iii) had sex with known HIV-positive primary sexual partner (as defined by the participant); (iv) had more than 2 concurrent sexual partners; (v) sexual intercourse when the participant or his partner were intoxicated; (vi) used illegal drugs (e.g., marijuana, dagga, heroin, ecstasy)
Promotional materials	HCD Flipchart and information, education and communication materials used in general VMMC demand creation activities
Mobilization platforms/channels/venues	Community engagement was targeted at population segments most likely to be at high risk of HIV exposure
	Mobilization conducted at targeted venues including at sugar plantations, fishing camps, taxi ranks, brothels, university/college campuses, bars, sports grounds, health clinics and pharmacies where men may seek treatment for STIs, and other late-night venues
Material- and non-material client compensation	*Phase 1* Clients did not receive financial compensation
	*Phase 2* ZMW 200 conditional on getting medically circumcised at one of the study sites within 3 months of enrolment
VMMC service delivery	Routine, comprehensive service delivery in accordance with national guidelines and global quality standards
Linkage strategies	Active follow-up of VMMC clients who were newly diagnosed with HIV was conducted for a period of 2 months to facilitate linkage and retention
	For those who presented for VMMC at a clinic and had STI symptoms, they were actively followed-up for a period of 1 month to facilitate completion of medications and VMMC surgery

Offering the financial compensation of ZMW 200 cash to clients was based upon prior research from Kenya [18], the minimum cost-of-living index in Zambia calculated for 3 days of work and travel costs for surgery and two follow-up visits.

### Study Procedures

Mobilizers recruited men aged ≥ 18 years at venues within the catchment area of the selected VMMC sites. Eligibility was determined through participant self-report of circumcision status, age and residency. If found to be eligible and interested, men were asked to provide written consent. After obtaining written consent, mobilizers administered the risk questionnaire. Men who were determined to be at high-risk (i.e., answer yes to at least one high risk question) were enrolled in the study. Once enrolled, in Phase 1 mobilizers conducted one-on-one interpersonal communication lasting 30–45 min using materials in the HCD flipchart and also completed other study questionnaires. In Phase 2, after completing the same study procedures as in Phase 1, participants were informed that they would receive ZMW 200 cash conditional on getting medically circumcised at one of the study sites within 3 months of enrolment. At the end of the session in both Phase 1 and 2 participants were provided with a referral voucher to be presented at the VMMC site within 3 months of enrolment.

At the VMMC site, when enrolled men presented the referral voucher to the program staff at the health facility, they were asked to complete the facility study questionnaire and then assessed for circumcision eligibility. If they had no contraindications, Phase 1 participants were surgically circumcised, and Phase 2 participants were surgically circumcised and given ZMW 200 within 5 days of circumcision. Phase 2 participants whose surgery was temporarily deferred (e.g., newly diagnosed HIV positive clients or STI symptoms) did not receive the payment unless they returned to the site after getting clearance from ART clinic or after treatment of the STI and got circumcised within 3 months of recruitment. Newly diagnosed HIV-positive clients were actively followed up by phone until they were linked and initiated ART.

### Data Analysis

The primary outcome for measuring the effectiveness of the enhanced demand creation and financial compensation strategy was the proportion of high-risk men aged ≥ 18 years undergoing VMMC at the participating facilities within 3 months of recruitment between the phases of the program. The generalized linear regression model was used to estimate effectiveness of intervention after adjusting for characteristics including number of risk factors reported, client age, and district of residence. Adjusted relative risks and prevalence estimates are presented with 95% confidence intervals (CI). The clustering at the level of mobilisers was accounted for using Huber White sandwich errors. Other outcomes of interest included proportion of high-risk men aged ≥ 18 years who were diagnosed with HIV or STI who initiate HIV or STI treatment through enhanced linkage. Statistical analyses were performed using Stata version 14 [24].

### Cost Assessment

Incremental costs of implementing enhanced demand creation strategies and financial compensation were assessed prospectively at the study sites for Phase 1 and Phase 2, respectively. Costs associated with the implementation of enhanced demand creation strategy and financial compensation were collected by relevant input type (personnel, travel, supplies, and compensation) over the project implementation period. Costs were collected from provider perspective using a combination of top-down and micro-costing approaches. Costs related to setting up and managing the project were excluded as these are considered non-program costs. Unit costs were estimated by dividing the total costs by the number of clients reached and the number of clients circumcised. Costs were presented in 2019 USD.

### Human Subjects

Ethical approval for this evaluation was obtained from ERES Converge IRB in Zambia (Ref# 2017-May-054), and The Johns Hopkins University School of Public Health Institutional Review Board (IRB# 00008078). This project was also reviewed in accordance with the Centers for Disease Control and Prevention (CDC) human research protection procedures and was determined to be research, but CDC investigators did not interact with human subjects or have access to identifiable data or specimens for research purposes. Written informed consent was obtained from all participants.

## Results

### Participant Description

A total of 9827 uncircumcised men, 18 years and older were enrolled into the study, 6096 in Phase 1 (June–Dec 2018) and 3731 in Phase 2 (Feb–March 2019). Enrollment for both phases was designed to run for 6 months, but Phase 2 enrollment was stopped after 7 weeks due to a rapid and significant increase in VMMC uptake which quickly depleted the funds budgeted for the incentives. Participants in both phases had similar demographic characteristics and no statistically significant differences were noted—over 70% of clients were 18–29 years old, 59% were never married, and almost all participants had a primary school education or higher. Overall, 74% of participants had a bank account (Table 2).

**Table 2:** Sociodemographic characteristics of participants (N = 9827)

	Phase 1 n (%)	Phase 2 n (%)	Total N (%)	Pearson chi-square (χ^2^) Design-based F	P value
*Age groups*					
18–24	3388 (56)	1973 (53)	5361 (55)	Uncorrected χ^2^ (6) = 14.1935 Design-based F (3.03, 75.63) = 0.8134	0.491
25–29	1230 (20)	747 (20)	1977 (20)		
30–34	680 (11)	458 (12)	1138 (12)		
35–39	360 (6)	275 (7)	635 (6)		
40–44	239 (4)	148 (4)	387 (4)		
45–49	116 (2)	81 (2)	197 (2)		
50+	83 (1)	49 (1)	132 (1)		
*Marital status*					
Never married	3640 (60)	2191 (59)	5831 (59)	Uncorrected χ^2^ (3) = 32.7073 Design-based F (2.05, 51.23) = 1.9193	0.156
Married/living together	2190 (36)	1451 (39)	3641 (37)		
Divorced/Separated	233 (4)	72 (2)	305 (3)		
Widowed	31 (1)	17 (0)	48 (0)		
*Education*					
Primary	1964 (32)	1221 (33)	3185 (32)	Uncorrected χ^2^ (2) = 47.2898 Design-based F (1.94, 48.54) = 1.0836	0.345
Secondary	3532 (58)	1983 (53)	5515 (56)		
Post-secondary	598 (10)	527 (14)	1125 (11)		
*Tribe*					
Bemba	1056 (17)	523 (14)	1579 (16)	Uncorrected χ^2^ (7) = 103.1440 Design-based F (2.27, 56.84) = 2.1684	0.117
Kaonde	60 (1)	21 (1)	81 (1)		
Lozi	534 (9)	328 (9)	862 (9)		
Lunda	38 (1)	18 (1)	56 (1)		
Luvale	86 (1)	24 (1)	110 (1)		
Ngoni	1020 (17)	480 (13)	1500 (15)		
Tonga	2421 (40)	1827 (49)	4248 (43)		
Other	879 (14)	510 (14)	1389 (14)		
*Assets*					
Bank account	4033 (66)	3257 (87)	7290 (74)	Uncorrected χ^2^ (1) = 538.9756 Design-based F (1.00, 25.00) = 11.8811	0.002
Agricultural land	2433 (40)	1349 (36)	3782 (38)	Uncorrected χ^2^ (1) = 13.8773 Design-based F (1.00, 25.00) = 0.2188	0.644
Livestock	2186 (36)	1306 (35)	3492 (36)	Uncorrected χ^2^ (1) = 0.7599 Design-based F (1.00, 25.00) = 0.0106	0.919

### Risk Factors

**Fig. 2:**
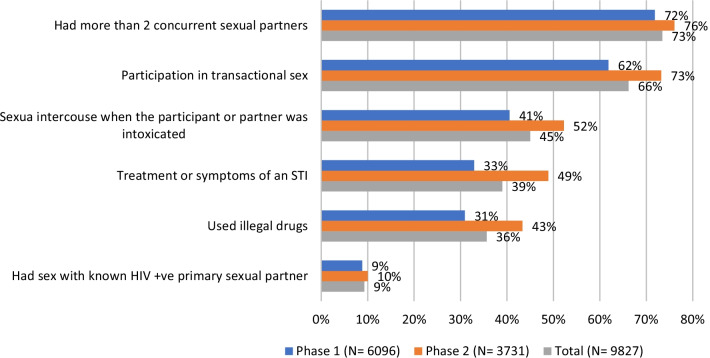
Self-reported HIV risk factors of enrolled study participants

Of the 9827 men enrolled in the evaluation, 7626 (78%) reported 2 risk factors or more. The most commonly reported risk behaviors were having more than 2 concurrent sexual partners (73%) and participation in transactional sex (66%) (Fig. 2). The reported risk level for participants increased in Phase 2, with more enrolled men self-reporting more risk factors in Phase 2 compared to Phase 1. Self-reported risk behaviors among circumcised clients were similarly distributed (not shown).

### VMMC Uptake

**Fig. 3:**
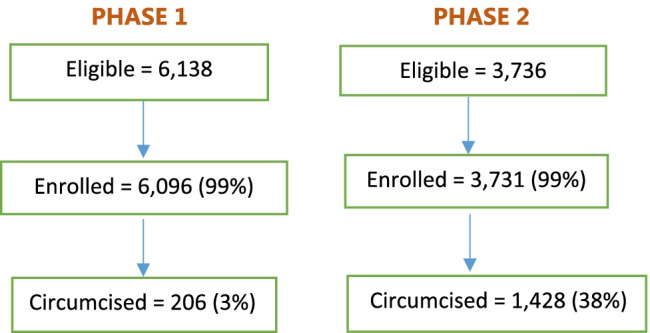
Study participants by phase (N = 9874)

Of the 6096 participants enrolled in Phase 1, 206 (3%) underwent VMMC. In the second Phase, 3731 men were enrolled, and 1428 (38%) underwent VMMC (Fig. 3). The proportion of men undergoing circumcision was significantly higher in Phase 2 compared to Phase 1 (38% vs 3%, uncorrected χ^2^ (1) = 2032.8906, F (1.00, 25.00) = 103.0866, *P* < 0.001).

Based on the generalized linear regression model (Table 3), Phase 2 enrollees were 8.8 times (RR 8.8, 95% CI − 3.6 to 21.6; *P* < 0.001) more likely to come for VMMC than Phase 1 enrollees and men older than 30 years of age, were 1.6 times (RR 1.6, 95% CI − 1.1 to 2.5; *P* = 0.026) more likely to come to the facility for VMMC than those aged less than 30 years. Men 25 years and older were 1.1 times more likely to go for VMMC with financial compensation compared to men 18–24 years old. Without compensation, these odds reduced to 0.87 times. In both phases, over 50% of participants who underwent circumcision were circumcised within 1 week of recruitment.

**Table 3:** Generalized linear regression tables

Circumcised	Risk ratio	P value	[95% conf. interval]	
Phase 1	Ref			
Phase 2	8.8	0.000	3.6	21.6
*Age*				
18–19 years	Ref			
20–29 years	0.9	0.813	0.6	1.5
30+ years	0.7	0.028	0.5	1.0
Phase 1*18–24 years	Ref			
Phase 1*25–50+ years	0.87	0.353	0.66	1.2
Phase 2*18–24 years	Ref			
Phase 2*25–50+ years	1.1	0.154	0.96	1.3
Interaction term for phase and age	Ref			
Phase 2*20–29 years	1.1	0.661	0.7	1.8
Phase 2*30+ years	1.6	0.026	1.1	2.5
Presence of bank account	1.4	0.043	1.0	1.9
Number of risk behaviors reported	1.0	0.456	0.9	1.1

Overall, 85% of clients who underwent circumcision were between 18 and 34 years of age and 61% had never been married (Table 4). In Phase 1, none of the participants who self-reported that they were very unlikely to get circumcised underwent circumcision. In Phase 2, 23% of clients who self-reported that they were very unlikely to get circumcised underwent circumcision. Over 90% of participants circumcised in Phase 2 reported that the cash financial compensation was very important and 98% said that the amount of compensation was just right or more than needed.

**Table 4:** Sociodemographic characteristics of circumcised participants (n = 1634)

	Phase 1 (%)	Phase 2 (%)	Total (%)	Pearson chi-square χ^2^ Design-based F	P value
*Age groups*					
18–19	46 (22)	238 (17)	284 (17)	Uncorrected χ^2^ (7) = 82.9068 Design-based F (4.21, 105.25) = 1.9787	0.100
20–24	75 (36)	496 (35)	571 (35)		
25–29	46 (22)	285 (20)	331 (20)		
30–34	24 (12)	186 (13)	210 (13)		
35–39	8 (4)	110 (8)	118 (7)		
40–44	4 (2)	54 (4)	58 (4)		
45–49	1 (0)	42 (3)	43 (3)		
50+	2 (1)	17 (1)	19 (1)		
*Marital status*					
Never married	135 (66)	867 (61)	1002 (61)	Uncorrected χ^2^ (3) = 31.5556 Design-based F (2.70, 67.60) = 0.8867	0.444
Married/living together	64 (31)	528 (37)	592(36)		
Divorced/Separated	7(3)	27 (2)	34 (2)		
Widowed	0	6	6 (1)		
*Education*					
Primary	49 (24)	397 (27)	428 (26)	Uncorrected χ^2^ (2) = 34.8064 Design-based F (1.67, 41.80) = 0.5772	0.536
Secondary	130 (63)	783 (55)	913 (56)		
Post-secondary	27 (13)	266 (19)	293 (18)		
*Tribe*					
Bemba	51 (25)	232 (16)	283 (17)	Uncorrected χ^2^ (7) = 317.3233 Design-based F (3.29, 82.15) = 4.0166	0.008
Kaonde	2 (1)	9 (1)	11 (1)		
Lozi	19 (9)	126 (9)	145 (9)		
Lunda	1 (1)	5 (1)	6 (1)		
Luvale	6 (3)	9 (1)	15 (1)		
Ngoni	47 (23)	189 (13)	236 (14)		
Tonga	45 (22)	641 (45)	686 (42)		
Other	35 (17)	217 (15)	252 (15)		
*Assets*					
Bank account	156 (76)	1284 (90)	1440 (88)	Uncorrected χ^2^ (1) = 208.2970 Design-based F (1.00, 25.00) = 3.7102	0.066
Agricultural land	71 (34)	543 (38)	14 (38)	Uncorrected χ^2^ (1) = 5.8472 Design-based F (1.00, 25.00) = 0.3877	0.539
Livestock	79 (38)	541 (38)	620 (38)	Uncorrected χ^2^ (1) = 0.0992 Design-based F (1.00, 25.00) = 0.0028	0.958

### Linkage to STI and HIV Treatment

Overall, the number of clients diagnosed with an STI and the number of clients testing positive for HIV were very low in both Phases. The linkage rate for clients with an STI was 100% (8 clients) in both Phases and the linkage rate for participants newly diagnosed HIV-positive to antiretroviral therapy (ART) was 83% (5 of 6 clients) in Phase 1 and 50% (3 of 6 clients) in Phase 2. These clients were linked to ART services within 2 months of enrollment. Clients who were not linked to ART services were actively followed up by phone for 2 months and declined to be linked to services or until they asked not to be contacted again.

### Cost Analysis

The estimated total program costs for Phases 1 and 2 were $37,126.87 and $53,234.21, respectively (Table 5). Since supplies were procured for use in both phases, supplies costs were allocated equally by month across two phases. For Phase 1, costs were incurred most for travel (39%) followed by personnel (31%) and supplies (30%). In Phase 2, financial compensation accounted over half of the total costs (57%) followed by travel (23%), personnel (13%) and supplies (7%). The estimated costs per clients reached were $6.05 in Phase 1 and $14.50 in Phase 2. Costs per client that received VMMC in Phase 1 and Phase 2 were $151.54 and $34.93, respectively.

**Table 5:** Total program costs by phase and input type and costs per client, 2019 USD

Input type	Phase 1		Phase 2	
	Total costs	%	Total costs	%
Personnel	$11,494.55	31	$6989.62	13
Supplies	$11,313.27	30	$3771.09	7
Travel	$14,319.04	39	$11,993.50	23
Financial compensation	–	–	$30,480.00	57
Total	$37,126.87	100	$53,234.21	100
Cost per client reached	$6.05	–	$14.50	–
Cost per client circumcised	$151.54	–	$34.93	–

## Discussion

The findings show that enhanced demand creation with financial compensation of ZMW 200 significantly increased the uptake of VMMC among high-risk adult men in Zambia compared with enhanced demand creation alone and that the effect is most pronounced in men aged 30 years and older. Although the VMMC uptake rate in Phase 1 with the enhanced demand creation alone was low (3%), the one-on-one HCD approach and risk screening at targeted venues identified high numbers of uncircumcised men at high-risk for HIV acquisition. Circumcising HIV-negative men aged 20–34 years at highest risk of HIV acquisition achieves the greatest effectiveness for VMMC programs [9, 10]; in this evaluation, most men undergoing circumcision were between 18 and 34 years old (85%) and reported at least 2 high-risk behaviors.

Overall, total costs for Phase 2 were higher than those in Phase 1 driven by provision of financial compensation for undergoing VMMC. Although Phase 2 was implemented for a shorter period and incurred overall higher costs driven by financial compensation, with the higher VMMC uptake rate costs per VMMC client in Phase 2 were lower. Therefore, adding the financial compensation to the enhanced demand creation package is more cost-effective than implementing demand creation activities alone. A mop-up approach by intensifying the resources for a short period of time like Phase 2 activities to complement the routine VMMC services may help maximize the impact of investment on VMMC program. Our findings supported the evidence from other studies and demonstrated that the appropriate size of incentive can make the VMMC program cost-effective [17].

This study had several limitations. First, the study did not ask questions on employment status, or wealth, preventing further analysis into the socioeconomic status of study participants to determine if employment or income played a role in VMMC uptake among those that had been offered the financial compensation for missed work. Second, because payments were made electronically via mobile money systems, poor internet connectivity in some rural areas led to challenges in transmission of mobile payments to clients. Participants who encountered challenges may have shared their experience with other participants which may have discouraged some from going for circumcision. Another limitation was the relatively short study period of 3 months to get circumcised after recruitment. Because the decision to get circumcised often takes much longer [25], it is possible that some of the study clients would ultimately undergo circumcision as a result of the intervention but following participants longer wasn’t feasible. Seasonality may also have affected results particularly in Mazabuka district—Phase 1 (June–December 2018) occurred during fishing and cane cutting season. Study progress reports from mobilizers describing challenges to recruitment revealed seasonal workers did not have employer approval to take time off work for an elective procedure, and Phase 1 overlapped with the cane cutting season. Conversely, Phase 2 coincided with a fishing ban and cane cutting down time, which may have contributed to workers’ interest and availability to participate in VMMC, irrespective of the offer of economic compensation. Therefore, results may artificially overstate the effect of economic compensation for seasonal workers as this population could access VMMC more conveniently in Phase 2 than in Phase 1 and that seasonal work fluctuations may influence VMMC uptake. Because the study did not ask employment questions, further analysis cannot be performed. Finally, an operational challenge occurred in Phase 2—word of mouth spread quickly that the study was compensating men for VMMC. This may have led to exaggerated reporting of personal risk-taking in order to get recruited into the study. Health promotors were trained to probe the potential participants’ responses in order to ensure that they had engaged in the risk behaviors, but it was possible that some participants intentionally misreported personal risk-taking. Secondly, some study participants who were recruited in Phase 1 but did not get circumcised may have tried to get enrolled in Phase 2 when they heard about the compensation. Because participants were enrolled using their national registration card (NRC) number as the unique identifier, it was not possible for a participant to enroll into the study multiple times, although there may have been some participants who could have used a different national registration number because the study did not ask participants to show proof of the NRC.

This study adds to the evidence in support of using financial compensation to increase the uptake of VMMC [11]. Similar to our findings, several studies evaluating the impact of economic compensation on VMMC uptake found that cash reimbursements for transportation and food vouchers to partially compensate for wage loss were effective at motivating older men who had already been interested in circumcision to get circumcised [15–17]. A systematic review of the effectiveness of demand creation interventions for VMMC in sub-Saharan Africa found that demand creation strategies offering financial incentives produced the greatest impact on VMMC uptake [11].

Several important factors will have to be considered as VMMC programs explore the use of financial compensation to increase VMMC uptake. First, programs will have to consider how to introduce compensation for VMMC in healthcare facilities where other health services do not compensate clients to receive services; secondly, setting compensation amounts that would not be viewed as coercion and will be in line with Ministry of Health and donor policies [12] and third, strict eligibility criteria for clients receiving financial compensation for missed work. As this study was designed to increase VMMC uptake among older, high-risk men, programs considering a similar approach could provide a cash financial compensation for a limited time period (mop-up approach), to high-risk, hard-to-reach clients who get circumcised within that time period.

## Conclusions

Enhanced demand creation of targeted messaging at non-traditional VMMC recruitment sites with financial compensation of ZMW 200 cash equivalent to 3 days of missed work and transport costs significantly and rapidly increased VMMC uptake among high-risk men aged 18 years and older compared with enhanced demand creation alone. This strategy was also found to be cost-effective with lower costs per VMMC client. Compensating men for financial loss due to missed work and transport costs when they undergo VMMC appears to be an effective strategy for increasing service uptake among high-risk men in Zambia. Other VMMC programs could consider a similar approach where demand among high-risk men has been low.

## References

[1] Auvert B, Taljaard D, Lagarde E, Sobngwi-Tambekou J, Sitta R, Puren A (2005). Randomized, controlled intervention trial of male circumcision for reduction of HIV infection risk: the ANRS 1265 Trial. PLoS Med.

[2] Bailey RC, Moses S, Parker CB, Agot K, Maclean I, Krieger JN, Williams CF, Campbell RT, Ndinya-Achola JO (2007). Male circumcision for HIV prevention in young men in Kisumu, Kenya: a randomised controlled trial. Lancet.

[3] Gray RH, Kigozi G, Serwadda D, Makumbi F, Watya S, Nalugoda F, Kiwanuka N, Moulton LH, Chaudhary MA, Chen MZ, Sewankambo NK, Wabwire-Mangen F, Bacon MC, Williams CF, Opendi P, Reynolds SJ, Laeyendecker O, Quinn TC, Wawer MJ (2007). Male circumcision for HIV prevention in men in Rakai, Uganda: a randomised trial. Lancet.

[4] World Health Organization (WHO) (2007). New data on male circumcision and HIV prevention: policy and program implications. WHO/UNAIDS technical consultation on male circumcision and HIV prevention: research implications for policy and programming.

[5] Joint United Nations Programme on HIV/AIDS (UNAIDS). Fast-track: ending the AIDS epidemic by 2030. Available from: https://www.unaids.org/sites/default/files/media_asset/JC2686_WAD2014report_en.pdf.

[6] Joint United Nations Programme on HIV/AIDS (UNAIDS)/World Health Organization (WHO). Voluntary medical male circumcision. Progress brief 2019. https://www.unaids.org/sites/default/files/media_asset/2019_vmmc-15-esa-countries_en.pdf.

[7] Zambia Ministry of Health (2019) Zambia Population-based HIV Impact Assessment (ZAMPHIA) 2016: Final Report. Ministry of Health, Lusaka.

[8] United Nations Joint Programme on HIV/AIDS (UNAIDS), Global AIDS update 2019—communities at the centre. Available from: https://www.unaids.org/sites/default/files/media_asset/2019-global-AIDS-update_en.pdf.

[9] Awad SF, Sgaier SK, Tambatamba BC, Mohamoud YA, Lau FK, Reed JB, Njeuhmeli E, Abu-Raddad LJ (2015). Investigating voluntary medical male circumcision program efficiency gains through subpopulation prioritization: insights from application to Zambia. PLoS ONE.

[10] Kripke K, Opuni M, Schnure M, Sgaier S, Castor D, Reed J (2016). Age targeting of voluntary medical male circumcision programs using the Decision Makers’ Program Planning Toolkit (DMPPT) 2.0. PLoS ONE.

[11] Ensor S, Davies B, Rai T, Ward H (2019). The effectiveness of demand creation interventions for voluntary male medical circumcision for HIV prevention in sub-Saharan Africa: a mixed methods systematic review. J Int AIDS Soc.

[12] Gold E, Carrasco MA, Sallet J, Njhoma C, Musiige A, Rozario A, Makokha M, Grove S. Guide on high-impact practices to create demand for voluntary medical male circumcision services. Strengthening high-impact interventions for an AIDS-Free Generation (AIDSFree) Project. 2019. Available from: https://www.malecircumcision.org/resource/guide-high-impact-practices-create-demand-voluntary-medical-male-circumcision-services.

[13] Carrasco MA, Wilkinson J, Kasdan B, Fleming P (2019). Systematic review of barriers and facilitators to voluntary medical male circumcision in priority countries and programmatic implications for service uptake. Glob Public Health.

[14] World Health Organization. Manual for male circumcision under local anaesthesia and HIV prevention services for adolescent boys and men. Geneva: World Health Organization; 2018. Licence: CC BY-NC-SA 3.0 IGO.

[15] Carrasco MA, Grund JM, Davis SM, Ridzon R, Mattingly M, Wilkinson J, Kasdan B, Kiggundu V, Njeuhmeli E (2018). Systematic review of the effect of economic compensation and incentives on uptake of voluntary medical male circumcision among men in sub-Saharan Africa. AIDS Care.

[16] Kennedy CE, Yeh PT, Atkins K, Fonner VA, Sweat MD, O'Reilly KR, Rutherford GW, Baggaley R, Samuelson J (2020). Economic compensation interventions to increase uptake of voluntary medical male circumcision for HIV prevention: a systematic review and meta-analysis. PLoS ONE.

[17] Choko AT, Candfield S, Maheswaran H, Lepine A, Corbett EL, Fielding K (2018). The effect of demand-side financial incentives for increasing linkage into HIV treatment and voluntary medical male circumcision: a systematic review and meta-analysis of randomised controlled trials in low- and middle-income countries. PLoS ONE.

[18] Thirumurthy H, Masters SH, Rao S, Bronson MA, Lanham M, Omanga E, Evens E, Agot K (2014). Effect of providing conditional economic compensation on uptake of voluntary medical male circumcision in Kenya: a randomized clinical trial. JAMA.

[19] Thirumurthy H, Masters SH, Rao S, Murray K, Prasad R, Zivin JG, Omanga E, Agot K (2016). The effects of providing fixed compensation and lottery-based rewards on uptake of medical male circumcision in Kenya: a randomized trial. J Acquir Immune Defic Syndr.

[20] Marshall E, Rain-Taljaard R, Tsepe M, Monkwe C, Taljaard D, Hlatswayo F, Xaba D, Molomo T, Lissouba P, Puren A, Auvert B (2017). Obtaining a male circumcision prevalence rate of 80% among adults in a short time: an observational prospective intervention study in the Orange Farm township of South Africa. Medicine (Baltimore).

[21] Wilson N, Frade S, Rech D, Friedman W (2016). Advertising for demand creation for voluntary medical male circumcision. J Acquir Immune Defic Syndr.

[22] Bazant E, Mahler H, Machaku M, Lemwayi R, Kulindwa Y, Gisenge Lija J, Mpora B, Ochola D, Sarkar S, Williams E, Plotkin M, Juma J (2016). A randomized evaluation of a demand creation lottery for voluntary medical male circumcision among adults in Tanzania. J Acquir Immune Defic Syndr.

[23] Sgaier SK, Eletskaya M, Engl E, Mugurungi O, Tambatamba B, Ncube G, Xaba S, Nanga A, Gogolina S, Odawo P, Gumede-Moyo S, Kretschmer S (2017). A case study for a psychographic-behavioral segmentation approach for targeted demand generation in voluntary medical male circumcision. eLife.

[24] StataCorp (2015). Stata statistical software: release 14.

[25] Price JE, Phiri L, Mulenga D, Hewett PC, Topp SM, Shiliya N, Hatzold K (2014). Behavior change pathways to voluntary medical male circumcision: narrative interviews with circumcision clients in Zambia. PLoS ONE.

